# Boerhaave Syndrome: A Case Report

**DOI:** 10.5334/jbsr.3823

**Published:** 2025-03-18

**Authors:** Marie Laure Gharingam, Axel Edouard Vanrossomme

**Affiliations:** 1 Service d’Imagerie Médicale du CHU Charleroi‑Chimay, Belgique

**Keywords:** Spontaneous esophageal rupture, Boerhaave syndrome, computed tomography

## Abstract

A case is presented of Boerhaave syndrome in a 70‑year‑old female patient complaining of dysphagia with solids for a long time. She consulted for abdominal pain for 24 hours, accompanied by nausea and fecal vomiting. The patient had tachypnea, tachycardia, hypoxia, abdominal guarding, and absence of bowel sounds. Thoracoabdominal computed tomography revealed significant pyloric wall thickening with secondary gastric distension, cervical subcutaneous emphysema, bilateral pleural effusion, and pneumomediastinum.

## Introduction

Boerhaave syndrome (BS) is a non‑traumatic rupture of the esophageal wall, typically occurring during forceful vomiting. Middle‑aged men are predominately affected, and the rupture usually occurs in the lower third of the esophagus. The clinical presentation may be atypical. Computed tomography (CT) has a crucial role in diagnosing and full assessment of the extent of the condition and complications. This report presents a case of BS followed by a comprehensive review of the literature.

## Case Report

**Figure 1:**
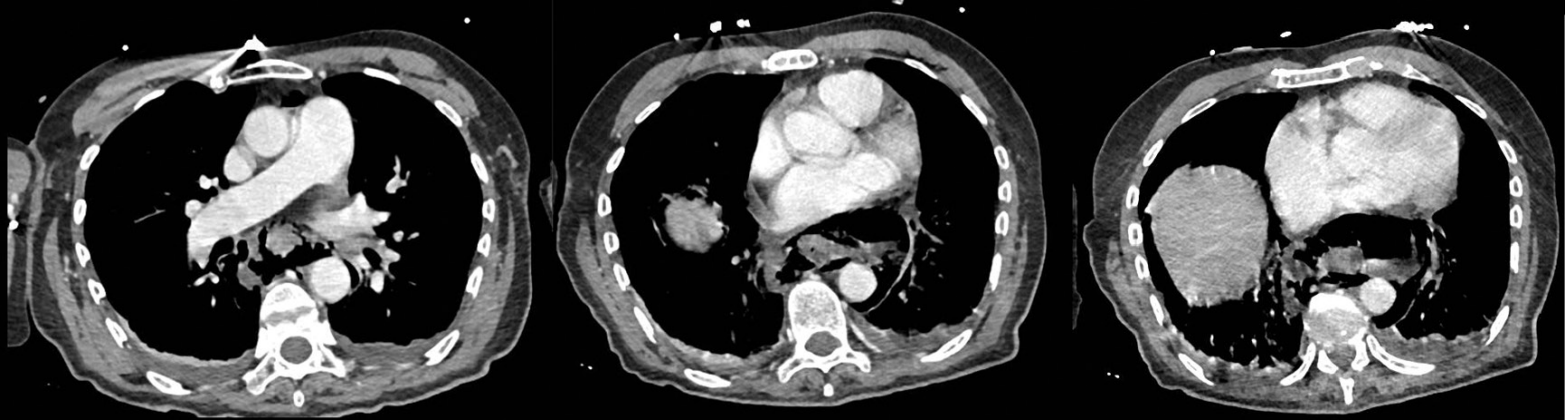
Contrast‑enhanced chest CT. Axial views in the parenchymal window show intramural hematoma, peri‑esophageal air collections indicating esophageal perforation, esophageal wall thickening, and bilateral pleural effusion. Source: Charleroi University Hospital.

**Figure 2:**
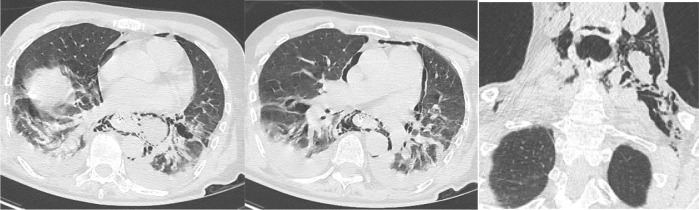
Chest CT. Axial views in the lung window and coronal reformatting demonstrating cervical subcutaneous emphysema and pneumomediastinum. Source: Charleroi University Hospital.

A 70‑year‑old woman was admitted with severe, sudden‑onset abdominal pain persisting for 24 hours, accompanied by nausea and fecaloid vomiting. Her medical history included sequelae of poliomyelitis and several months of dysphagia with solids. Clinically, the patient had tachypnea, tachycardia, hypoxia, abdominal guarding with absence of bowel sounds, and cognitive impairment. Laboratory tests revealed marked leukocytosis. Thoracoabdominal computed tomography (CT) revealed substantial pyloric wall thickening with secondary gastric distension, cervical subcutaneous emphysema, bilateral pleural effusion, and pneumomediastinum (Figures 1 and 2). The diagnosis of BS was suggested as the primary condition.

**Figure 3:**
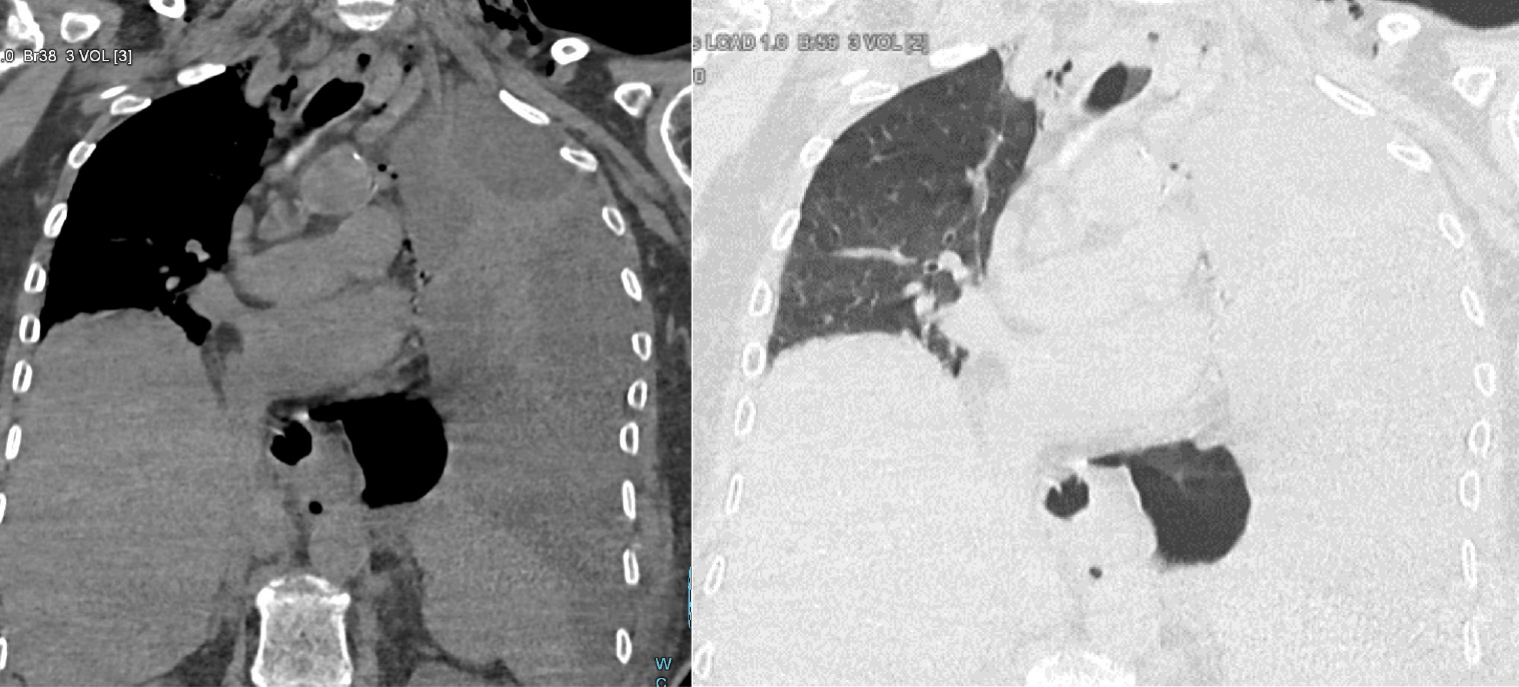
Follow‑up chest CT. Coronal reformatting in the mediastinal and pulmonary windows showing encapsulated pleural effusion (empyema) and complete left lung atelectasis. Source: Charleroi University Hospital.

Given the patient’s critical condition and medical history, neither gastroscopy nor surgical intervention could be performed. A multidisciplinary team comprising emergency, visceral surgery, and intensive care specialists opted for supportive care, including nutritional support and antibiotic therapy. Unfortunately, the patient’s condition deteriorated, characterized by a progressive pneumomediastinum and a left‑sided pleural effusion, which evolved into empyema with complete left lung atelectasis (Figure 3).

Her clinical evolution was further marked by mediastinitis and acute respiratory failure and, likely, superior vena cava syndrome. Despite the prompt and coordinated efforts of the multidisciplinary team, the patient died a few days later.

## Discussion

Digestive perforation is a clinical condition with an estimated incidence of 3.1 per million, predominantly resulting from iatrogenic causes [1, 2, 3, 5]. BS is a less common cause of esophageal perforation [3, 5]. BS is characterized by a complete transmural rupture of the esophagus and should be distinguished from Mallory–Weiss syndrome, which involves partial esophageal tears [1]. The rupture is caused by a sudden and significant increase in intra‑esophageal pressure during vomiting [1, 2]. It most commonly occurs in the posterolateral aspect of the distal thoracic esophagus, often on the left side. This predisposition is due to the relatively thin muscle fibers and lack of local protective structures [1, 3].

The diagnosis of BS is often associated with Mackler’s triad, which includes lower chest pain, vomiting, and cervical subcutaneous emphysema [3, 5]. In advanced stages, Gott’s tetrad, characterized by lower thoracic pain accompanied by vomiting and hematemesis, cervical subcutaneous emphysema, respiratory distress, and prostration, may be observed [2]. A male predominance is reported, with a significant history of heavy alcohol consumption (40%) and neurological disorders (10%) [2, 3].

CT has a reported sensitivity and specificity of 92–100% in identifying esophageal perforation and associated mediastinitis [5]. Classically, pneumomediastinum, left‑sided pleural effusion, left‑sided pneumothorax, and air around the esophagus may be observed. Cervical and thoracic subcutaneous emphysema may also occur [5].

Boerhaave syndrome is challenging in both diagnosis and management [2]. Non‑surgical management may be considered on the basis of the patient’s comorbidities [3]. Risk classification using the Pittsburgh score (low, medium, and high risk) contributes to tailoring treatment to the individual patient [5]. With mortality rates reaching up to 90%, early diagnosis, ideally within the first 12 hours post‑rupture, and prompt multidisciplinary management are essential for improving patient outcomes [5].

## Conclusion

Spontaneous esophageal perforation, also known as Boerhaave syndrome, is a rare, yet life‑threatening, condition characterized by high morbidity and mortality rates.
